# Approaches to modernize the combination drug development paradigm

**DOI:** 10.1186/s13073-016-0369-x

**Published:** 2016-10-28

**Authors:** Daphne Day, Lillian L. Siu

**Affiliations:** 1 Drug Development Program, Division of Medical Oncology & Hematology, Princess Margaret Cancer Centre, Toronto, Ontario M5G 2M9 Canada; 2 Department of Medicine, University of Toronto, Toronto, Ontario M5S 1A8 Canada; 3 OICR Research Fellow, Ontario Institute for Cancer Research, Toronto, Ontario M5G 0A3 Canada

## Abstract

Recent advances in genomic sequencing and omics-based capabilities are uncovering tremendous therapeutic opportunities and rapidly transforming the field of cancer medicine. Molecularly targeted agents aim to exploit key tumor-specific vulnerabilities such as oncogenic or non-oncogenic addiction and synthetic lethality. Additionally, immunotherapies targeting the host immune system are proving to be another promising and complementary approach. Owing to substantial tumor genomic and immunologic complexities, combination strategies are likely to be required to adequately disrupt intricate molecular interactions and provide meaningful long-term benefit to patients. To optimize the therapeutic success and application of combination therapies, systematic scientific discovery will need to be coupled with novel and efficient clinical trial approaches. Indeed, a paradigm shift is required to drive precision medicine forward, from the traditional “drug-centric” model of clinical development in pursuit of small incremental benefits in large heterogeneous groups of patients, to a “strategy-centric” model to provide customized transformative treatments in molecularly stratified subsets of patients or even in individual patients. Crucially, to combat the numerous challenges facing combination drug development—including our growing but incomplete understanding of tumor biology, technical and informatics limitations, and escalating financial costs—aligned goals and multidisciplinary collaboration are imperative to collectively harness knowledge and fuel continual innovation.

## Background

The principle underlying combining therapeutic agents is to maximize efficacy and overcome treatment resistance by utilizing drugs with known activity, different mechanisms of action, and minimally overlapping toxicities. Cytotoxic chemotherapy combinations have had an indispensable impact in oncology and malignant hematology. Indeed, almost all curative cytotoxic regimens consist of combination agents [1]. Many of these combinations were discovered in a “trial and error” or empirical manner, often with limited nonclinical data of synergism.

In the past two decades, our growing genomic knowledge underlying oncogenesis has shifted the focus of developmental therapeutics to molecularly targeted agents (MTAs). This shift is coupled with advances and increasing availability of next-generation sequencing and other novel molecular techniques such as transcriptome analysis, RNA interference screening, and genome-editing tools. MTAs aim to optimize the therapeutic index by exploiting key tumor-specific vulnerabilities such as oncogenic or non-oncogenic addiction and synthetic lethality (Box 1). However, substantial genomic complexity exists, such that tumors are rarely reliant on one molecular aberrant pathway for survival, which, with a few notable exceptions, limits the efficacy and durability of response to single agent MTAs [2–4]. Beyond MTAs, immuno-oncology agents have produced impressive and durable tumor responses by reactivating host immunity and are approved for a growing number of indications, with combined immuno-oncology therapy showing enhanced antitumor activity in some cases [5–11]. Furthermore, emerging evidence suggests an interplay between the tumor genomic landscape and immune response, providing a rationale for the therapeutic integration of immune-based and genomically based strategies [12–17].

As in the case of cytotoxics, combinatorial approaches are needed for MTAs and immuno-oncology agents to adequately disrupt intricate molecular and immune interactions to provide long-term clinical benefit. However, progress in this field is hampered by a multitude of challenges. Foremost among these is the rational selection of combinations in the perplexing and dynamic disease context, which is characterized by tumor genomic redundancy and adaptability and considerable intra- and inter-patient heterogeneity [18, 19]. Secondly, clinical trial methodology is not optimized for the evaluation of MTA and immuno-oncology combinations and novel approaches are urgently needed. Thirdly, concerted efforts from regulatory authorities, investigators, and pharmaceutical companies are crucial to enable efficient drug discovery and development.

This review summarizes some of the past successes and failures in the development of combination therapies, explores the obstacles ahead, and suggests future directions to manage the evolving dynamics of cancer.

## Past and present status of combination drug development

### Types of MTA combinations

MTAs can be combined to inhibit multiple components within a signaling network to evade resistance mechanisms or to target distinct and potentially complementary oncogenic processes. Combination strategies may include (1) additive or synergistic drug combinations of the same mechanism or connected mechanisms of action, (2) synthetic lethality pairings, and (3) the addition of a second agent with a different mechanistic activity to reverse resistance mechanisms. In addition, MTAs can be combined with other therapeutic modalities, such as radiotherapy, chemotherapy, and immuno-oncology therapy. Table 1 demonstrates some examples of these approaches.

**Table 1:** Types of combinations

Types of combinations	Examples
(1) Synergistic or additive combinations	
Targeting the same molecule for maximal target inhibition	Dual human epidermal growth factor receptor 2 (HER2) blockade (pertuzumab and trastuzumab in *HER2*-amplified breast cancer) [23, 25]
Vertical targeting: inhibiting two or more targets along the same pathway	BRAF and MEK inhibition (vemurafenib and cobimetinib, dabrafenib, and trametinib in melanoma) [27, 29]
Horizontal targeting: inhibiting parallel or compensatory pathways	Phosphoinositide 3-kinase (PI3K) and MEK inhibition (BKM120 and trametinib in *RAS*- or *BRAF*-mutant solid tumors) [128]
(2) Synthetic lethality	Poly(ADP-ribose) polymerase (PARP) inhibitor and DNA-damaging agent (veliparib plus platinum-based chemotherapy in triple-negative breast cancer) (NCT02032277)
(3) Reversal of resistance	Cyclin-dependent kinase (CDK) and estrogen receptor (ER) inhibition (palbociclib and fulvestrant in hormone-receptor-positive breast cancer in postmenopausal women) [30]

### Approved MTA combinations

Between January 2006 and June 2016, four MTA–MTA and four MTA–endocrine-therapy combinations were approved by the US Food and Drug Administration (FDA) for use in adult solid malignancies, compared with approximately 40 approved single-agent MTAs and approximately 20 MTA–chemotherapy combinations (Table 2) [20, 21]. These combination approvals are based on randomized phase III or phase II trial data demonstrating improved progression-free survival or overall survival compared with the established standard of care, which is almost always one of the agents in the combination with or without chemotherapy [22–30]. In all cases, one or both drugs were FDA approved prior to being approved as a combination for the same disease indication.

**Table 2:** FDA approvals of MTA or immuno-oncology combinations in adult solid tumors between January 2006 and June 2016 [20]

Year of approval	Tumor type	Combination^a^	Biomarker(s)
2016	RCC	Lenvatinib + everolimus^b^	
2016	Breast	Palbociclib + fulvestrant^b^	HR positive, HER2-negative
2015	Squamous NSCLC	Necitumumab + cisplatin/gemcitabine	
2015	Melanoma	Cobimetinib + vemurafenib^b^	*BRAF* V600 mutation
2015	Melanoma	Nivolumab + Ipilimumab^b^	
2015	CRC	Ramucirumab + FOLFIRI	
2015	Breast	Palbociclib + letrozole^b^	HR positive, HER2-negative
2014	NSCLC	Ramucirumab + docetaxel	
2014	Ovarian, fallopian tube, primary peritoneal	Bevacizumab + paclitaxel, liposomal doxorubicin or topotecan	
2014	Cervix	Bevacizumab + paclitaxel/cisplatin or paclitaxel/topotecan	
2014	Gastric/GE junction	Ramucirumab + paclitaxel	
2014	Melanoma	Trametinib + dabrafenib^b^	*BRAF* V600 mutation
2012	CRC	Ziv-aflibercept + FOLFIRI	
2012	Breast	Everolimus + exemestane^b^	HR positive, HER2-negative
2012	CRC	Cetuximab + FOLFIRI	*KRAS* wild type
2012	Breast	Pertuzumab + trastuzumab and docetaxel^b^	*HER2* amplified/protein overexpression
2011	SCCHN	Cetuximab + platinum/fluoropyrimidine	
2010	Gastric/GE junction	Trastuzumab + cisplatin/fluoropyrimidine	HER2 protein overexpression
2010	Breast	Lapatinib + letrozole^b^	*HER2* amplified/protein overexpression and HR positive
2009	RCC	Bevacizumab + interferon-α	
2008	Breast	Bevacizumab + paclitaxel	HER2 negative
2007	Breast	Lapatinib + capecitabine	*HER2* amplified/protein overexpression
2006	Breast	Trastuzumab + AC–T	*HER2* amplified/protein overexpression
2006	NSCLC	Bevacizumab + platinum-based chemotherapy	
2006	CRC	Bevacizumab + fluoropyrimidine-based chemotherapy	
2006	SCCHN	Cetuximab + radiation	

^a^Expanded indications in the same tumor type are not listed again in this table

^b^MTA–MTA, MTA–endocrine therapy or immuno-oncology–immuno-oncology combinations

*AC–T* doxorubicin/cyclophosphamide–paclitaxel, *CRC* colorectal cancer, *FOLFIRI* fluorouracil/leucovorin/irinotecan, *GE* gastro-esophageal, *HR* hormone receptor, *MTA* molecularly targeted agent, *NSCLC* non-small-cell lung cancer, *RCC* renal cell carcinoma, *SCCHN* squamous cell carcinoma of the head and neck

In addition to the MTA–MTA and MTA–endocrine-therapy combinations, ipilimumab and nivolumab are two immuno-oncology agents also approved as a doublet regimen. Rather than targeting aberrant genomic pathways, these monoclonal antibodies (mAbs) inhibit immune regulatory checkpoints, cytotoxic T-lymphocyte-associated antigen 4 (CTLA-4) and programmed cell death protein-1 (PD-1), respectively, producing durable tumor regression in multiple tumor types [5–11]. Mechanistically, combined CTLA-4 and PD-1 blockade demonstrated enhanced treatment efficacy by targeting non-redundant immune pathways [31, 32].

The scientific basis of these nine FDA-approved combinations is founded on proof of resistance mechanisms to an established therapy and/or evidence of synergistic or additive activity in animal models [28, 33–42]. The targeting of the mitogen-activated protein kinase (MAPK) pathway at two key levels is an example of using dual targeted therapy to effectively counteract genetic escape mechanisms. In the treatment of advanced malignant melanoma, combined inhibition of BRAF and its downstream effector MAPK kinase (MEK) led to improved survival outcomes compared with BRAF inhibition alone. The doublet regimen prevents MAPK pathway activation, which is the most common mechanism of acquired resistance to BRAF inhibitors [27, 29, 38, 39, 43, 44]. Notably, in these nine approved combinations, MTAs are used at or near their single-agent recommended dose, without substantial increase in toxicity. Additionally, in seven out of the nine combinations—with the exceptions of lenvatinib and everolimus, and nivolumab and ipilimumab—established predictive biomarkers are utilized for molecularly based patient selection [22–30].

### Lessons learnt from unsuccessful MTA combinations

Approximately 75 % of investigational oncology compounds that enter clinical testing do not ultimately receive regulatory approval; these include 50 % of drugs tested in the phase III setting [45]. In most of these cases, investigators could not have predicted the negative results, and explanations for the lack of efficacy are often deficient. In Table 3, we highlight some of the potential reasons underlying past failed drug combinations.

**Table 3:** Challenges of combination drug development and examples of unsuccessful combinations

	Challenges	Examples
Target validity and engagement	• Discordance between nonclinical and clinical data • Difficulty characterizing biological relevance and functionality of the target(s), target engagement and modulation by the investigational agent(s), and pathway interactions due to absent or poorly designed pharmacodynamics studies in early clinical studies	Selumetinib (MEK inhibitor) + MK-2206 (AKT inhibitor) in metastatic CRC (phase II) • Promising nonclinical data • Target inhibition not consistently reached • Other potential reasons for failure include possible activation of compensatory mechanisms and overlapping toxicities [129]
Pharmacological effect of drug combination	• Effect of drug combinations, which may be additive, synergistic, or antagonistic, has a direct impact on antitumor activity and toxicity • Often poorly understood in both nonclinical and clinical environments	Adjuvant tamoxifen + anthracycline-based chemotherapy in breast cancer found to be inferior to sequential tamoxifen following chemotherapy (phase III) • Antagonistic effect suggested by nonclinical data [130, 131]
Patient selection	• Being able to accurately select the subgroup of patients who would derive maximal benefit can substantially broaden the therapeutic window. However, identification, validation, and standardization of predictive biomarkers remain very difficult	IMC-A12, R1507 or CP-751,871 (IGF-1R inhibitors) + erlotinib (EGFR inhibitor) in metastatic NSCLC in three separate trials (phase I/II, phase II and phase III, respectively) • Promising nonclinical data • Three negative studies with limited activity in unselected patients • No biomarker identified from the studies • Also poor tolerance seen [132–134]
Toxicity	• Poor drug tolerance affects the maintenance of dose intensity and duration, thereby limiting efficacy, particularly if two agents share the same target or have overlapping side effects • Small-molecule TKI combinations may be more likely than mAb combinations to cause increased off-target toxicities and pharmacokinetic interactions via the cytochrome P450 system [65]	Four phase I studies • Ipilimumab (CTLA-4 inhibitor) + vemurafenib (BRAF inhibitor) led to hepatotoxicity [135] • Tremelimumab (CTLA-4 inhibitor) + sunitinib (VEGFR inhibitor) led to renal toxity [136] • Bevacizumab (VEGF inhibitor) + sunitinib led to vascular/hematological toxicities [137] • Temsirolimus (mTOR inhibitor) + sunitinib led to skin/hematological toxicities [138]

*CRC* colorectal cancer, *mAb* monoclonal antibody, *NSCLC* non-small-cell lung cancer, *TKI* tyrosine kinase inhibitor

Failure to show benefit at the phase III stage is particularly disappointing during drug development given the immense financial cost and human resources involved. Importantly, a large number of patients may have been exposed to harm or unnecessary treatment. An example is the combination of anti-epidermal growth factor receptor (EGFR) antibodies (cetuximab or panitumumab), anti-vascular endothelial growth factor (VEGF) antibody (bevacizumab), and chemotherapy in metastatic colorectal cancer. Both anti-EGFR and anti-VEGF mAbs have established antitumor activity in combination with chemotherapy in this setting [46–48]. A large body of nonclinical data demonstrated synergism produced by combined EGFR and VEGF blockade and a small phase II study in irinotecan-refractory patients demonstrated clinical benefit [49–51]. However, in two phase III trials, which together included over 1700 patients, the addition of cetuximab or panitumumab to bevacizumab and chemotherapy in the frontline setting unexpectedly resulted in significantly shorter progression-free survival, including in the *KRAS* wild-type subpopulation, and toxicity was also increased [52, 53]. The reasons behind this discouraging outcome are not known. Discontinuation rates secondary to toxicity were similar in both arms in one study [53]. The investigators postulated that unfavorable pharmacokinetic and pharmacodynamic interactions between the anti-EGFR and anti-VEGF antibodies may have occurred, leading to the blunting of the therapeutic effect of each agent [52, 53]. This example serves as a reminder that although combining drugs with proven mechanisms of action is an attractive and logical strategy, carefully designed early clinical trials with comprehensive pharmacokinetic and pharmacodynamic parameters are essential to understand drug interactions and to provide proof of concept.

## Nonclinical development

### Selecting and prioritizing combinations: a systematic approach to drug discovery

Currently, there are approximately 100 approved anticancer drugs and, according to the Pharmaceutical Research and Manufacturers of America’s 2015 report, approximately 1200 new anticancer drugs are in development in the United States, amounting to approximately 845,000 possible pairings and an exponential number of higher-order combinations [54]. This, in addition to the expanding number of potential drug targets, necessitates systematic and efficient methods of drug discovery.

Increasingly, industry and academia are utilizing various methods of high-throughput screening, which leverage laboratory automation to simultaneously assay the biological activities of a vast number of compounds [55–59]. Indeed, unbiased chemical screening may uncover unexpected interactions, likely attributable to previously unknown interconnected cellular signaling pathways [60, 61]. For example, in an attempt to identify therapeutic partners for the Bruton’s tyrosine kinase (BTK) inhibitor ibrutinib, a high-throughput screen study in diffuse large B-cell-lymphoma cell-line models demonstrated impressive combinatorial activity with a range of mechanistically distinct drug classes, which may warrant further investigation [55]. Additional technological advances include in silico modeling methods to facilitate large-scale genome-wide identification of candidate synthetically lethal genes as new drug targets and to predict drug response [62]. Computational network-based algorithms can also systematically analyze gene regulatory and signaling pathways to mechanistically define genetic determinants of disease and establish new therapeutic targets [63]. Furthermore, ex vivo testing in cell culture models derived from patient samples with acquired resistance may prove to yield more robust and predictive tumor models than existing models for therapeutic testing [64].

Complementary to these novel approaches, data-sharing efforts are imperative to promote scientific collaboration. Examples include publically available data repositories that catalogue protein–protein interactions and biological pathways such as Pathway Commons and Database of Interacting Proteins [65, 66]. The US National Cancer Institute (NCI) recently launched Genomic Data Commons, an interactive data-sharing platform enabling the import and harmonization of genomic data from multiple research programs using standardized bioinformatics pipelines [67, 68]. Additionally, large annotated cell-line drug-screen libraries, such as the Cancer Cell Line Encyclopedia, Genomics of Drug Sensitivity in Cancer Project, and NCI60 (the US National Cancer Institute 60 human tumor cell line anticancer drug screen), are publically accessible datasets correlating drug sensitivity with detailed genomic data and thus serve as rich resources for researchers [69–74].

### Quality of nonclinical data

Nonclinical studies of drug combinations often report synergy without appropriate evaluation of the combination index (Box 1) [75]. Additionally, low rates (11–25 %) of reproducibility of published laboratory data, including those from high-impact journals, have been identified, despite attempts to recreate the experimental environment, suggesting limitations in the validity of scientific findings and probable publication bias [76, 77]. Improving the reliability and predictive value of nonclinical studies is fundamental to successful clinical translation. Box 2 outlines important issues to consider when designing the aims, experimental conditions, and parameters of nonclinical studies. At a minimum, benchmarks for nonclinical studies prior to consideration of clinical testing should include validation of a robust scientific hypothesis via demonstration of mechanism of action, observation of objective synergistic or additive antitumor activity, and acceptable safety at clinically achievable drug concentrations.

## Clinical development

In response to the fundamental challenges of tumor molecular diversity and the expanding portfolio of novel MTA and immuno-oncology agents, clinical trial designs and statistical approaches are evolving to optimize the evaluation of combination therapeutics and streamline their clinical development. Despite considerable progress, more innovative precision medicine-based approaches are urgently needed to implement customized treatments and to bring truly durable benefit for molecularly stratified subsets of patients or even for individual patients.

### Novel dose-finding strategies

Traditional rule-based dose-escalation trial designs, which rely on the premise of dose-dependent toxicity, may have considerable limitations in defining the biological optimal dose and schedule of combination MTAs. Unlike cytotoxic chemotherapy, the relationship between dose, toxicity, target inhibition, and efficacy is less predictable with MTA therapy [78]. Pharmacological interactions of drug combinations can also impact on dose effect. Furthermore, the assessment of dose-limiting toxicity (DLT), a key determinant of the maximum tolerated dose (MTD) and the recommended phase II dose, can be problematic for phase I MTA trials. The traditional DLT window of observation—typically the first cycle of treatment—used in trials of cytotoxic agents may not be adequate, as MTAs may display delayed toxicity due to their relatively long half-life and chronic dosing schedule. There is also a lack of consensus on the definitions of DLT, and these have been found to be widely heterogenous across MTA clinical trials [79].

Adaptive Bayesian model-based designs may be better placed to cater for the complex variables associated with combination MTAs, by incorporating pre-study probability of toxicity and updating such probability with real-time adverse event data to inform dose-escalation decisions [80–83]. In simulation studies, adaptive designs were found to maximize the number of patients treated at or near the MTD compared with rule-based designs [84, 85]. Importantly, adaptive designs allow prospective modifications on aspects of the trial as the data evolve, offering greater flexibility to researchers. Additionally, multiple methods have been proposed using both toxicity and efficacy as endpoints [86–88]. For example, the zone method describes a parallel phase I/II design that uses initial rule-based dose escalation and subsequent Bayesian adaptive randomization to enable simultaneous evaluation of the safety and efficacy of multiple dose combinations, such that sample size can be reduced and more patients are treated with the higher-efficacy dose levels [86]. However, one challenge of adaptive designs is the requirement for continual biostatistical modeling, which may have affected their uptake in the past, although the application of these designs is increasing with time [84, 89, 90]. An operationally hybrid approach is the modified toxicity probability interval design, which obviates the requirement for computational modeling by utilizing an up-and-down dose-assignment scheme conceptually similar to a rule-based algorithm but guided by Bayesian models [91, 92]. This design has been used in a number of phase I clinical trials [93, 94].

Currently, there is no preferred dose-escalation design for combination MTAs. The choice of the most appropriate dose schedule selection and dose-finding method should be informed by knowledge of the nonclinical and clinical pharmacology of the agents of interest and based on consultation between experienced clinical researchers, sponsors, and statisticians. Comprehensive pharmacokinetic evaluation and pharmacodynamic assessment of tumors in early phase trials are vital to assess for target modulation and to mechanistically characterize on- and off-target toxicities. These are particularly pertinent in combination studies to delineate individual drug effects and to evaluate pharmacological interactions. Multiple targets may also need to be cross-examined to assess network inter-dependencies in identifying resistance mechanisms.

### Integrating the immune landscape

Combinations of novel immune checkpoint and co-stimulatory molecules are actively being evaluated for additive or synergistic effects. Additionally, as multiple oncogenic pathways can foster immunosuppressive microenvironments, immuno-oncology agents are also being investigated in combination with MTAs, with pending efficacy data [12–17]. Although validated predictive biomarkers are presently lacking, emerging immune monitoring techniques are giving insights into the interactions between tumor antigen profiles, the microenvironment, and the immune response, guiding potentially personalized immuno-oncology strategies [95]. However, the unique properties of immunotherapies present multiple challenges to clinical trial design, demanding careful forethought on dosing and schedule decisions, patient selection, toxicity assessment, pharmacokinetic/pharmacodynamic monitoring, and choice of endpoints. For example, immuno-oncology agents often do not reach a MTD in dose-escalation studies and exhibit distinctive patterns of tumor response and immune-related toxicities, which are not necessarily dose-dependent. Furthermore, nonclinical studies of immuno-oncology agents are technically difficult owing to species-specific differences in immune response and the lack of reliable models [96].

### Improving the efficiency of drug development: a shift from the traditional three-phase approach

The desire to expedite drug development has challenged the conventional three-phase clinical trial paradigm, in which safety and efficacy objectives are mandated at each distinct stage to fulfill regulatory requirements. Indeed, the time period from first-in-human testing to regulatory approval for oncology drugs typically averaged 7–9 years, substantially longer than agents of other therapeutic classes [45, 97]. Novel designs including seamless phase I/II, II/III clinical trials and large dose-expansion cohorts in phase I trials are increasingly utilized to streamline clinical development, obviating lengthy pauses between trials [98–100]. For example, the anti-PD-1 mAb pembrolizumab received accelerated approval by the FDA in September 2014 for use in metastatic melanoma, based on efficacy data from 173 patients enrolled onto the expansion cohort of a phase Ib trial (KEYNOTE-001), a mere 3 years after clinical development began [8]. Dose-expansion cohorts are often conducted in phase I trials for anticancer agents given alone or in combination that demonstrate compelling early signals of activity. These “tails” in phase I trials, typically in the form of disease-specific or biomarker-specific cohorts, apply intermediate endpoints, such as objective response rate, to support accelerated approval [99]. However, the use of these strategies requires clearly defined goals, pragmatic protocols, and statistical design with the flexibility to respond to evolving data and objectives, independent oversight, and a commitment from researchers, commercial sponsors, and regulators to work in tandem [99]. Getting these factors right is important to strike a balance between protection of patient safety and interests and therapeutic innovation.

### Targeting tumor heterogeneity: genomically informed clinical trial designs and biomarker development

**Fig. 1:**
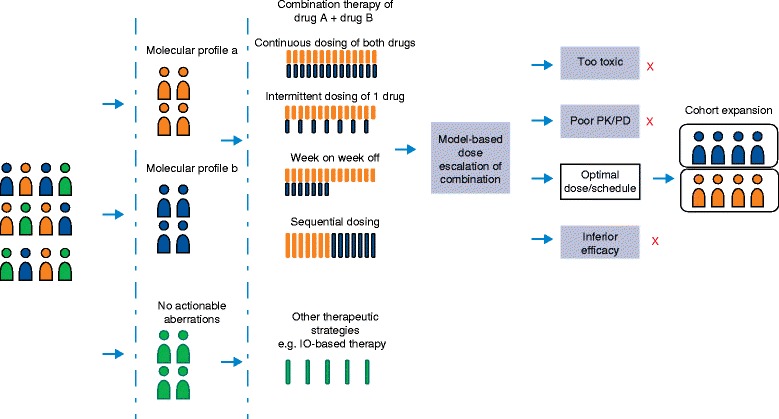
An example of an adaptive trial design. Patients are matched to treatments according to molecular subtype. Multiple doses and schedules are tested in dose escalation for the combination of drugs A and B. Adaptive randomization can be used to maximize the number of patients randomized to the most effective arm. Schedules that show inferior activity, inferior pharmacokinetic/pharmacodynamic profiles, or increased toxicity are stopped early (*red crosses*) and the most optimal dose/schedule is taken forward to cohort expansion. *IO* immuno-oncology, *PD* pharmacodynamics, *PK* pharmacokinetics

Multiple umbrella (histology-specific) and basket (histology-agnostic, aberration-specific) clinical trials are currently ongoing, incorporating genomic testing results for treatment assignment to genotype-matched therapies and combinations (Lung-MAP, NCT02154490; BATTLE, NCT00409968; NCI-MATCH, NCT02465060; and My Pathway, NCT02091141) (Box 1) [101]. The size and scope of these umbrella and basket trials can vary based on complexities and heterogeneities of tumor genotypes under evaluation. In some cases, adaptive Bayesian model-based designs can be embedded in these clinical trials to dynamically test multiple hypotheses, doses and schedules (Fig. 1). The adaptive design allows prospective decisions to be made based on accumulating results that become available during the course of the trial, such that more patients can be enrolled in cohorts showing the strongest efficacy signals and poorly performing cohorts can be closed early; and additional treatment cohorts testing novel agents and combinations may also be added. Although the clinical utility of genotype-matched trials remains to be proven, they provide a framework to efficiently probe the relationship between molecular aberrations, tumor histology, and MTA activity. Genotype-based trials may also be distinctly advantageous in matching targeted treatments for patients with low prevalence mutations, although extensive screening efforts or parallel molecular screening programs will be required to identify these patients. The establishment of national or international registries of patients whose tumors have undergone molecular profiling that enables rapid identification of rare genotypes might be useful. An additional challenge is that most of these molecularly based clinical trials rely on genomic sequencing data from a single tumor sample, offering only a static “snapshot” of patients’ genomic profiles. Cancer Research UK’s TRACERx trial sets a precedent for a prospective non-interventional study using multiregional and longitudinal tumor and circulating biomarker sampling to map the impact of dynamic intra-tumor heterogeneity during the course of disease progression. Insights gained from this study will inform the future development of dynamic therapeutic genomic trials [102].

To support molecular-based patient selection strategies, biomarker development and validation are crucial, preferably in concert with drug discovery and testing. A successful example is the development of crizotinib in anaplastic lymphoma kinase fusion gene (*ALK*)*-*translocated non-small cell lung cancer, in which the identification of a molecular subset of patients helped accelerate drug registration [4]. The currently ongoing I-SPY 2 (NCT01042379) is an adaptive multi-cohort trial for locally advanced breast cancer, aiming to identify biomarker-matched MTAs and MTA combinations. So far, three experimental therapies have demonstrated improved activity compared with standard therapy in distinct biomarker signature populations and have met pre-specified criteria for testing in confirmatory phase III trials [103–105]. In a recent review, biomarkers that aid patient selection were found to improve the phase transition probabilities of oncology drugs, although the majority of non-orphan drugs are still being developed without markers [106]. The US Institute of Medicine has released ten recommendations for the clinical development and use of biomarker tests, in recognition of the wide-ranging obstacles in the context of tumor heterogeneity, substantial technical difficulties in assay reproducibility and standardization, and reimbursement barriers [107]. Challenges notwithstanding, drug–biomarker pairing is vital to enhance the therapeutic index of MTAs and bring maximal benefit to the appropriate target population.

### Precision medicine: the individualized dynamic model

In addition to spatial tumor heterogeneity, there is increasing awareness of tumor clonal evolution as a key mechanism of therapeutic failure, whereby genomic and epigenetic alterations and resistant variants develop and proliferate under selective treatment pressures [108, 109]. Thus, effective precision medicine would need not only to respond to the molecular diversity unique to each individual patient but also to adapt to the evolving dynamics of cancer. The individualized dynamic model may be a solution to this complex challenge, allowing intelligent drug combinations to be tailored to the genomic and immune profile of individual patients. Critical to this approach is the longitudinal monitoring of the changing molecular landscape to assess for treatment efficacy, to enable the early discovery of emerging resistant clones, and to target these pre-emptively with new drugs or combinations prior to the onset of clinical or radiological progression.

To facilitate the collection of dynamic molecular information, novel techniques such as serial measurements of circulating tumor DNA (ctDNA) or cell-free DNA (cfDNA) can be used at key treatment time points or regular intervals and present a less invasive alternative to tumor biopsies [110, 111]. A recent study of serial cfDNA sampling utilizing next-generation sequencing in phase I patients demonstrated the feasibility of this approach and suggested that cfDNA allele frequency dynamics may correlate with clonal response to targeted therapy [112]. Moreover, a number of other approaches are showing early promise to assist with dynamic therapeutic monitoring and prediction of specific treatment susceptibilities. For example, patient-derived xenografts using patients’ own avatars for drug sensitivity testing may help to predict the emergence of resistance clones ex vivo and inform therapeutic options, although clinical application requires successful engraftment and timely generation of models [113, 114]. The use of patient-derived organoids may provide a suitable alternative with a faster turnaround time. Emerging radiomic techniques, which enable high-throughput extraction of a large number of quantitative features from imaging modalities (computed tomography (CT), positron emission tomography (PET), or magnetic resonance imaging (MRI)), bring hope of providing noninvasive methods to track phenotypic changes in anatomical imaging during treatment, and associations with underlying gene expression patterns have been shown [115, 116].

**Fig. 2:**
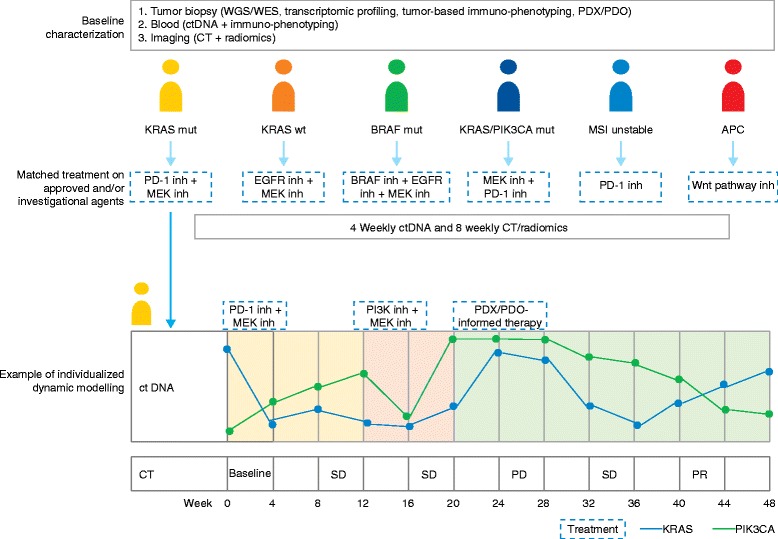
A proposed individualized dynamic study in colorectal cancer. Multiple hypotheses are tested in this parallel individualized dynamic design. This hypothetical example is in colorectal cancer patients after progression on standard therapies. Baseline tumor characterization includes whole-genome sequencing (WGS)/whole-exome sequencing (WES) and transcriptome sequencing from fresh tumor biopsies, circulating tumor DNA (ctDNA) sampling, immune profiling, and radiomics analysis. Patient-derived xenografts (PDXs)/patient-derived organoids (PDOs) are also generated. Drug therapy is then tailored to each patient’s mutational and immune profile. While on treatment, serial ctDNA sampling occurs 4 weekly and radiomics is performed every 8 weeks to guide therapeutic decisions. Patient one is used as an example: (1) at week 0, started on programmed cell death protein-1 (PD-1) inhibitor and MEK inhibitor; (2) at week 12, treatment is changed to phosphoinositide 3-kinase (PI3K) inhibitor and MEK inhibitor due to the increase in the allele frequency of a *PIK3CA* mutation; and (3) at week 20, the allele frequencies of both *PIK3CA* and *KRAS* mutations continue to rise and treatment is changed to therapy informed by PDX/PDO data. *CT* computed tomography, *mut* mutation, *PD* progressive disease, *PR* partial response, *SD* stable disease, *wt* wild type, *MSI* microsatellite instability, *inh* inhibitor

The proposed individualized dynamic model offers the compelling potential to deliver immediate and durable benefit to patients, as well as an opportunity to study disease evolution and biology at an individual genetic level. However, the implementation and scaling-up of this approach may face a myriad of technical, resource, and culture issues. In Table 4, key considerations in designing and executing dynamic genomics trials are highlighted. Figure 2 presents an example of an individualized dynamic trial design.

**Table 4:** Key components of individualized dynamic studies

Key components	Comments
Molecular and immune profiling at baseline	• Whole-exome or whole-genome sequencing, ideally using fresh tumor biopsies • Transcriptomic profiling • Proteomic validation • Immuno-phenotyping • Parallel ctDNA/cfDNA • PDX or PDO
Dynamic monitoring of molecular and immune landscapes	• Serial tumor biopsies may trigger concerns of safety and may not capture spatial heterogeneity • Novel strategies include serial monitoring of ctDNA/cfDNA, molecular imaging, and radiomics evaluation • ctDNA/cfDNA may assist with monitoring of treatment efficacy and early detection of resistant clones, although whether circulatory biomarkers reflect spatial tumor heterogeneity remains to be addressed • Assays need to have fast turnaround to allow “real-time” decision-making
Correlation of molecular monitoring with radiological response	• Requires exploration in future studies • Radiological response may not always provide the full picture, as in the case of mixed responses, and may lag behind treatment resistance • Also, pseudoprogression may occur in some cases with immuno-oncology agents
Multidimensional treatment algorithms at key decision points in response to molecular results	• If multiple mutations are present, treatment prioritization is required. Considerations may include relevance and level of evidence for the actionability of the mutation(s): that is, “driver” versus “passenger” mutations; allele frequency of mutation(s), and copy number change in the case of amplifications; downstream and parallel pathway aberrations that may confer treatment resistance; and availability of drugs and drug combinations. Sequential or alternating approaches may also be considered • Evaluation of immunotherapeutic approaches in cases of high mutational burden or other immune biomarkers to assess their predictive role • With the detection of emerging clones, consider changing therapeutic strategy ahead of radiological progression
Access to approved and investigational agents	• Requires collaboration with industry and academic partners

*ctDNA* circulating tumor DNA, *cfDNA* cell-free DNA, *PDO* patient-derived organoid, *PDX* patient-derived xenograft

## Addressing industry and regulatory barriers

A review of the characteristics of combination clinical trials listed on ClinicalTrials.gov between 2008 and 2013 found that 25.6 % of oncology trials were combination trials and, surprisingly, the ratio of combination trials to all trials decreased over time (*p* < 0.05), from 29.5 % in 2008 to 22.7 % in 2012. Furthermore, trials supported by the US National Institutes of Health are significantly more likely to use combinations than those supported by industry [117]. Barriers to industry investment and collaborations in combination therapy may include concerns regarding rising expenditure, intellectual property protection, toxicity and risk attribution, profit implications, and more complex regulatory pathways. However, industry alliances are vital to maximize the access of experimental therapy for nonclinical and clinical evaluations. Academia and cooperative groups may play a central unifying role. The NCI, for example, launched the Critical Molecular Pathways pilot project and developed template data sharing and intellectual property language for combination studies [72, 118]. The Institute of Medicine also sponsored a workshop to set standards on the application of models of precompetitive collaboration to align competing goals and facilitate industry-wide productivity [119]. Precompetitive collaboration refers to united efforts between companies to share the burden of research tasks for mutual benefit, often in early stages of product management, such as the development of common infrastructure and aggregation of data [119]. This may become a necessity in combination drug development owing to increasing biological complexity coupled with high rates of clinical failure. Additionally, commercial incentives for collaboration to develop combination treatments include the opportunity to re-purpose and market unsuccessful drugs, while reducing duplication of investigational pipelines. Recently, inter-company and industry–academia partnerships seem to have been invigorated, as evidenced by large genomically based trials such as NCI-MATCH, I-SPY 2, and Lung-MAP and the AstraZeneca–Sanger Institute Drug Combination Prediction DREAM Challenge [120].

Cumbersome clinical trial operational systems can substantially hinder and add to the cost of drug development. A study found that opening a phase III cooperative group trial required a median of 2.5 years from the time of concept review by the cooperative groups to trial opening at individual cancer centers [121]. The time to activation—the period from when a trial is submitted for consideration until it opens for enrolment—at cancer centers was median 120 days (range 21–836 days) [121]. Moreover, a direct statistical relationship was found between lengthy trial development and poor accrual in a linked study [122]. Thus, efforts should be directed at re-engineering and simplifying current processes for trial pre-activation, activation, and conduct, and, where possible, using central infrastructure and eliminating overlapping administrative and logistical requirements [123]. The Novartis Signature Program is an example of a basket trial with no pre-designated study sites, which utilizes a standard contract, budget, informed consent, and ethics process to rapidly open the study at institutions once a patient has been identified from local genomic profiling results [124].

The limited utility of single agents provides the impetus to combine drugs early in their development, rather than delaying until one or both drugs is approved. In recognition of this, the FDA published their guidance on the co-development of two or more new investigational drugs in 2013, which emphasizes the need for a biological rationale for early co-development and outlines recommendations for nonclinical and clinical testing. It also provides direction for approval and marketing processes, with an emphasis on encouraging early and regular dialogue between commercial sponsors and the FDA to streamline and purpose-fit their efforts [125]. These guidelines will complement existing expedited access programs—such as breakthrough designation, accelerated, and priority review—to assist with efficient combination therapy development.

## Conclusions

To adequately address the immense complexity and heterogeneity underlying oncogenesis and disease progression, innovative combination strategies will need to be customized to patients’ unique molecular and immune profiles and adaptively applied to respond to evolving changes over time. Moreover, in light of the current pace of scientific discovery and mounting financial costs, it is apparent that the existing framework of oncological drug development, with substantial attrition and lengthy timelines, is inefficient and ultimately unsustainable. Systematic high-throughput methods and computational network-based platforms can be utilized to explore novel therapeutic targets and identify synergistic or additive drug combinations. Clinical trial designs should be informed by comprehensive understanding of tumor biology and pharmacology and should leverage novel approaches to more effectively investigate new drug combinations. Throughout the nonclinical and clinical processes, co-development of biomarkers must be prioritized to refine and optimize patient selection. Importantly, meaningful collaborations and coordination of efforts are crucial among all stakeholders to collectively overcome technical, informatics, and logistical challenges, toward the shared goal of precision medicine.

## Box 1. Glossary terms (in order of appearance in text)

**Table:** 

Therapeutic index:	This describes the margin of safety of a drug. It is defined as the ratio of the dosage of a drug that produces toxicity in 50 % of subjects to the dose that produces the desired treatment effect in 50 % of subjects (TD_50_/ED_50_). Drugs with narrow or low therapeutic index are drugs with small differences between therapeutic and toxic doses.
Oncogene addiction:	A concept describing the dependence of cancer cells on the activity of an oncogene for survival. The inhibition of the oncogene may lead to cell death or arrest. For example, the *BCR-ABL* fusion oncogene, targeted by imatinib, is a major driver of tumorigeneis in chronic myelogenous leukemia [2].
Non-oncogene addiction:	Aside from oncogenes, tumorigenesis is reliant on a range of other genes and pathways. These non-oncogenes may be exploited as drug targets. An example is antiangiogenic therapy using VEGF inhibitors in renal cell carcinoma.
Synthetic lethality:	Two genes are said to be synthetically lethal if simultaneous loss of function of both genes results in cellular death but the loss of function of either gene leads to a viable phenotype. An example is the selective susceptibility to PARP inhibition in *BRCA1/BRCA2*-deficient cells [126].
Combination index:	This quantitatively describes combination drug interactions, where a combination index (CI) < 1 indicates a greater effect than the expected additive effect (synergism), CI = 1 indicates a similar effect (additive), and CI > 1 indicates a lesser effect (antagonism).
Umbrella trial:	Genotype-based clinical trials testing different drugs matched to molecular aberrations in a single cancer type. An example is the Lung-MAP trial (NCT02154490) in patients with squamous non-small cell lung cancer, which investigates multiple therapies matched to specific molecular aberrations.
Basket trial:	Genotype-based clinical trials testing one or more drugs targeting one or more molecular aberrations in a variety of cancer types. A single trial may involve multiple cohorts, which are generally defined by cancer type. An example is a clinical trial of vemurafenib, a BRAF inhibitor in multiple non-melanoma cancers with *BRAF* V600 mutations [127].

## Box 2. Suggestions for improving the quality of nonclinical studies

**Table:** 

Suggestions		Benefits
Use multiple cell lines and animal models with molecular characterization		To recapitulate tumor heterogeneity and the influence of host effects
Characterize pharmacokinetic and pharmacodynamic interactions		To reach an understanding of the interactions between drugs, their targets, and the downstream effects
Study optimal concentration and exposure of each drug for target engagement		To inform the dosing ratio and schedule to be explored in clinical trials
Identify biomarkers to be further explored and refined in early phase trials		To assist with patient selection or stratification
Set a predetermined benchmark prior to contemplating clinical testing		To reduce the chance of futile clinical trials

## Abbreviations

AC–T: Doxorubicin/cyclophosphamide–paclitaxel
cfDNA: Cell-free DNA
CRC: Colorectal cancer
CT: Computed tomography
ctDNA: Circulating tumor DNA
FDA: Food and Drug Administration
FOLFIRI: Fluorouracil/leucovorin/irinotecan
GE: Gastro-esophageal
HR: Hormone receptor
inh: Inhibitor
IO: Immuno-oncology
mAB: Monoclonal antibody
MRI: Magnetic resonance imaging
MSI: Microsatellite instability
MTA: Molecularly targeted agents
MTD: Maximum tolerated dose
mut: Mutation
NCI: National Cancer Institute
NSCLC: Non-small cell lung cancer
PD: Pharmacodynamics
PDO: Patient-derived organoid
PDX: Patient-derived xenograft
PET: Positron emission tomography
PK: Pharmacokinetics
RCC: Renal cell carcinoma
SCCHN: Squamous cell carcinoma of the head and neck
TKI: Tyrosine kinase inhibitor
WES: Whole-exome sequencing
WGS: Whole-genome sequencing
wt: Wild type
